# Methyl 3,5,5,6,8,8-hexa­methyl-5,6,7,8-tetra­hydro­naphthalene-2-carboxyl­ate (AHTN–COOMe)

**DOI:** 10.1107/S1600536811002601

**Published:** 2011-01-26

**Authors:** Paul Kuhlich, Franziska Emmerling, Christian Piechotta, Irene Nehls

**Affiliations:** a BAM Federal Institute for Materials Research and Testing, Richard-Willstätter-Strasse 11, D-12489 Berlin-Adlershof, Germany

## Abstract

Crystals of the title compound, C_18_H_26_O_2_, were grown from ethyl acetate. Due to the racemic precursor, the title compound is also obtained as a racemate. Disorder was observed during structure refinement, originating from two possible half-chair conformations of the non-aromatic ring. The disorder was refined by introducing split positions in the cyclo-hexane ring regarding the two possible *R* and *S*-enantiomers at the chiral CH group [ratio 0.744 (3):0.256 (3)]. The crystal structure features pairs of inversion-related molecules connected by pairs of non-classical C—H⋯O hydrogen bonds.

## Related literature

For the occurrence of the title compound in human breast milk and the fatty tissue of fish, see: Valdersnes *et al.* (2006 ▶). The title compound is the product of an esterification of 3,5,5,6,8,8-hexa­methyl-5,6,7,8-tetra­hydro­naphthalene-2-carb­oxy­lic acid (AHTN—COOH) with methanol. For the synthesis of the acid, see: Kuhlich *et al.* (2010 ▶); Valdersnes *et al.* (2006 ▶). For the crystal structures of AHTN and AHTN–COOH, see: De Ridder *et al.* (1990 ▶) and Kuhlich *et al.* (2010 ▶), respectively. For the environmental occurrence and estrogenic activity of AHTN, see: Heberer (2003 ▶); Bitsch *et al.* (2002 ▶). For puckering parameters, see: Cremer & Pople (1975 ▶).
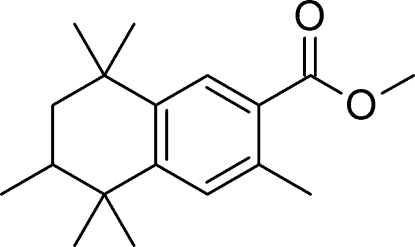


## Experimental

### 

#### Crystal data

C_18_H_26_O_2_
*M* *_r_* = 274.39
Monoclinic, 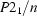

*a* = 11.5049 (11) Å
*b* = 11.9482 (5) Å
*c* = 12.1078 (13) Å
β = 102.612 (5)°
*V* = 1624.2 (2) Å^3^
*Z* = 4
Cu *K*α radiation
μ = 0.55 mm^−1^
*T* = 193 K
0.45 × 0.40 × 0.30 mm

#### Data collection

Enraf–Nonius CAD-4 diffractometer
5083 measured reflections
3062 independent reflections
2856 reflections with *I* > 2σ(*I*)
*R*_int_ = 0.047
3 standard reflections every 60 min  intensity decay: 3%

#### Refinement

*R*[*F* ^2^ > 2σ(*F* ^2^)] = 0.054
*wR*(*F* ^2^) = 0.152
*S* = 1.03
3062 reflections
216 parameters
10 restraints
H-atom parameters constrained
Δρ_max_ = 0.29 e Å^−3^
Δρ_min_ = −0.26 e Å^−3^

### 

Data collection: *CAD-4 Software* (Enraf–Nonius, 1989 ▶); cell refinement: *CAD-4 Software*; data reduction: *CORINC* (Dräger & Gattow, 1971 ▶); program(s) used to solve structure: *SIR97* (Altomare *et al.*, 1999 ▶); program(s) used to refine structure: *SHELXTL* (Sheldrick, 2008 ▶); molecular graphics: *PLATON* (Spek, 2009 ▶); software used to prepare material for publication: *PLATON*.

## Supplementary Material

Crystal structure: contains datablocks I, global. DOI: 10.1107/S1600536811002601/zl2346sup1.cif

Structure factors: contains datablocks I. DOI: 10.1107/S1600536811002601/zl2346Isup2.hkl

Additional supplementary materials:  crystallographic information; 3D view; checkCIF report

## Figures and Tables

**Table 1:** Hydrogen-bond geometry (Å, °)

*D*—H⋯*A*	*D*—H	H⋯*A*	*D*⋯*A*	*D*—H⋯*A*
C18—H26⋯O2^i^	0.98	2.47	3.397 (2)	157

Symmetry code: (i) 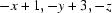.

**Fig. 1.**
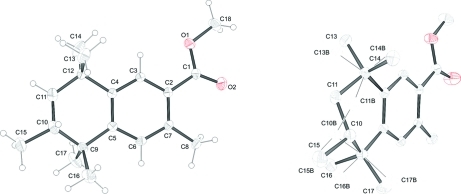
: Left: ORTEP representation of the title compound with atomic labeling shown with 30% probability displacement ellipsoids. Right: Atoms belonging to the minor disordered moiety and their bonds are presented in light gray and as thin black lines respectively. Hydrogen atoms are omitted for the sake of clarity.

**Fig. 2.**
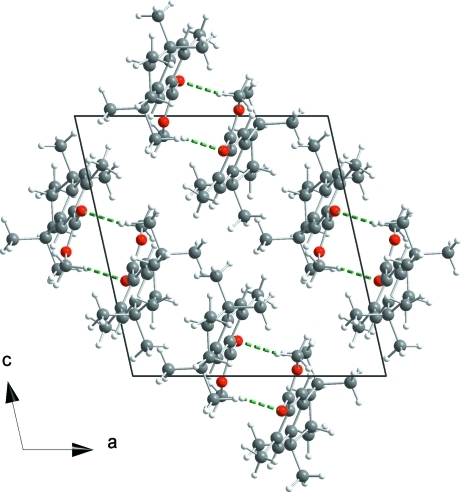
: View of the unit cell of the title compound along [010]. Hydrogen bonds are drawn as dashed green lines. The minor disordered moiety is omitted for clarity.

## supplementary crystallographic information

### Comment

The title compound is the product of an esterification of 3,5,5,6,8,8-hexamethyl-5,6,7,8- tetrahydronaphthalene-2-carboxylic acid (AHTN-COOH) with methanol. AHTN-COOH itself is the product of a haloform reaction from 1-(3,5,5,6,8,8-hexamethyl-5,6,7,8- tetrahydronaphthalen-2-yl)ethan-1-one (AHTN) and sodium hypochlorite solution. Two slightly different syntheses of AHTN-COOH are described by Kuhlich *et al.* (2010) and by Valdersnes *et al.* (2006). The crystal structure of AHTN-COOH was described previously by Kuhlich *et al.* (2010). The crystal structure of AHTN was determined by De Ridder *et al.* (1990).

The title compound can either be obtained in a two-step synthesis, described by Valdersnes *et al.* (2006) or, as described here, in a one-step procedure (see Experimental).

AHTN itself is a widely used fragrance in cosmetics and cleaning products. It is introduced into the environment mainly via sewage treatment plants and can be found in surface water at low µg/*L* concentration (Heberer, 2003). It is in focus of interest due to its low estrogenic potential (Bitsch *et al.*, 2002). Due to their structural similarities to AHTN, the title compound and AHTN-COOH might also have estrogenic or even toxic properties themselves.

The title compound was found in human breast milk and piscine fatty tissue by Valdersnes *et al.* (2006) proofing its ubiquitary occurrence.

The molecule crystallizes in the monoclinic space group *P*2_1_/n. The molecular structure of the compound and the atom-labeling scheme are shown in Fig 1. The structure is disordered in the non aromatic ring. This disorder can be described as pseudo mirror-symmetric with respect to the aromatic ring's plane, resulting in two moieties (ratio 0.744 (3):0.256 (3)).

A general puckering analysis according to Cremer and Pople (Cremer & Pople, 1975) led to a half-chair conformation for both enantiomers. The S-enantiomer (C4-C5-C9-C10B-C11B-C12) has a puckering amplitude (Q) of 0.526 (6) Å and 0.502 (2) Å for the R-enantiomer (C4-C5-C9-C10-C11-C12), respectively. The maximum deviation from planarity for C11/C11B is -0.3587 (39) of the R-enantiomer and 0.3335 (14) of the S-enantiomer, respectively, proofing the nearly mirror-symmetric setup.

Each molecule is surrounded by three next neighbors, whereas the centroids of the molecules are arranged in sheets parallel to the (202) plane.

A detailed description of the disorder treatment can be found in the refinement section. The molecules form pairs *via* non classical hydrogen bonds (C18—H26···O2) (see dashed green bonds in Fig. 2).

### Experimental

The methyl ester of 1-(3,5,5,6,8,8-hexamethyl-5,6,7,8- tetrahydronaphthalen-2-yl)ethan-1-one (AHTN) was synthesized by stirring a solution of 1 mg AHTN dissolved in 100 mL methanol and 50 mL 10% sodium hypochlorite solution at room temperature. After 24 h of stirring precipitated sodium chloride was dissolved with water and the organic compound was extracted three times with 50 mL ethyl acetate each. The organic solvents were combined, washed with water and dried with sodium sulfate. For single-crystal X-ray crystallography white and clear crystals of the title compound were grown by solvent evaporation (ethyl acetate) at ambient temperature over a period of three days [m.p. 348 K]. IR (ν, cm^-1^): 1720(*s*), 1687(*s*), 1609(*s*), 1550(*s*), 1495(*s*), 1435(*s*), 1390(*s*), 1360(*s*), 1298(*s*), 1255(*s*), 1245(*s*), 1191(*s*), 1141(*s*), 1103(*s*), 1021(*s*), 980(*s*), 944(*s*), 916(*s*); ^1^H-NMR (500 MHz, CD_3_OD, TMS): δ = 7.83 (1*H*, s), 7.23 (1*H*, s), 3.83 (3*H*, s), 2.48 (3*H*, s), 1.85 (1*H*, ddq, *J*_H,H'_=2.7 Hz, *J*_H,H''_=13.2 Hz, *J*_H,Me_=6.9 Hz), 1.61 (1*H*, dd, ^2^*J*=13.6 Hz, ^3^*J*=13.2 Hz), 1.38 (1*H*, dd, ^2^*J*=13.6 Hz, ^3^*J*=2.7 Hz), 1.30 (3*H*, s), 1.26 (3*H*, s), 1.22 (3*H*, s), 1.04 (3*H*, s), 0.98 (3*H*, d, *J*=6.9 Hz); ^13^C-NMR (125 MHz, CD_3_OD, TMS): δ = 169.7, 151.8, 143.5, 137.8, 131.8, 130.2, 127.8, 52.1, 44.6, 38.9, 35.7, 34.9, 32.6, 32.4, 28.9, 25.1, 21.7, 17.1; (+)-ESI/MS: 275.6 (60) [*M*+H^+^], 297.5 (100) [*M*+Na^+^].

### Refinement

The structure exhibits disorder originating from two possible half chair conformations in the non aromatic ring. The significant disorder around the atoms of the non aromatic ring was taken into account and the refinement was improved by introducing split positions for the atoms C10, C11, C13, C14, C15, C16 and C17. Equivalent bond distances within the two moieties were restrained to be the same within a standard deviation of 0.02Å, and equivalent disordered atoms were constrained to have identical ADPs. Refinement of the occupancy ratio converged to a value of 74.4 (3)% for the major and 25.6 (3)% for the minor moiety, respectively.

Hydrogen atoms were placed in calculated positions with C—H distances of 0.98 (CH_3_), 0.99 (CH_2_), 1.00 (CH_sat_), and 0.95 (CH_arom_) with *U*_iso_(H) = 1.2 of the parent atom *U*_eq_ or 1.5 *U*_eq_(C_methyl_).

### Crystal data

**Table:**

C_18_H_26_O_2_	*F*(000) = 600
*M**_r_* = 274.39	*D*_x_ = 1.122 Mg m^−^^3^
Monoclinic, *P*2_1_/*n*	Cu *K*α radiation, λ = 1.54178 Å
Hall symbol: -P 2yn	Cell parameters from 25 reflections
*a* = 11.5049 (11) Å	θ = 65–69°
*b* = 11.9482 (5) Å	µ = 0.55 mm^−^^1^
*c* = 12.1078 (13) Å	*T* = 193 K
β = 102.612 (5)°	Block, colourless
*V* = 1624.2 (2) Å^3^	0.45 × 0.40 × 0.30 mm
*Z* = 4	

### Data collection

**Table:**

Enraf–Nonius CAD-4 diffractometer	*R*_int_ = 0.047
Radiation source: rotating anode	θ_max_ = 69.8°, θ_min_ = 4.8°
graphite	*h* = 0→14
ω/2θ scans	*k* = −14→14
5083 measured reflections	*l* = −14→14
3062 independent reflections	3 standard reflections every 60 min
2856 reflections with *I* > 2σ(*I*)	intensity decay: 3%

### Refinement

**Table:**

Refinement on *F*^2^	Secondary atom site location: difference Fourier map
Least-squares matrix: full	Hydrogen site location: inferred from neighbouring sites
*R*[*F*^2^ > 2σ(*F*^2^)] = 0.054	H-atom parameters constrained
*wR*(*F*^2^) = 0.152	*w* = 1/[σ^2^(*F*_o_^2^) + (0.0889*P*)^2^ + 0.4305*P*] where *P* = (*F*_o_^2^ + 2*F*_c_^2^)/3
*S* = 1.03	(Δ/σ)_max_ = 0.001
3062 reflections	Δρ_max_ = 0.29 e Å^−^^3^
216 parameters	Δρ_min_ = −0.26 e Å^−^^3^
10 restraints	Extinction correction: *SHELXTL* (Sheldrick, 2008), Fc^*^=kFc[1+0.001xFc^2^λ^3^/sin(2θ)]^-1/4^
Primary atom site location: structure-invariant direct methods	Extinction coefficient: 0.0070 (8)

### Special details

**Table:**

Geometry. All esds (except the esd in the dihedral angle between two l.s. planes) are estimated using the full covariance matrix. The cell esds are taken into account individually in the estimation of esds in distances, angles and torsion angles; correlations between esds in cell parameters are only used when they are defined by crystal symmetry. An approximate (isotropic) treatment of cell esds is used for estimating esds involving l.s. planes.
Refinement. Refinement of F^2^ against ALL reflections. The weighted R-factor wR and goodness of fit S are based on F^2^, conventional R-factors R are based on F, with F set to zero for negative F^2^. The threshold expression of F^2^ > 2sigma(F^2^) is used only for calculating R-factors(gt) etc. and is not relevant to the choice of reflections for refinement. R-factors based on F^2^ are statistically about twice as large as those based on F, and R- factors based on ALL data will be even larger.

### Fractional atomic coordinates and isotropic or equivalent isotropic displacement parameters (Å^2^)

**Table:**

	*x*	*y*	*z*	*U*_iso_*/*U*_eq_	Occ. (<1)
O1	0.34558 (10)	1.32637 (9)	−0.02324 (9)	0.0454 (3)	
O2	0.44327 (15)	1.40647 (10)	0.13483 (11)	0.0711 (5)	
C1	0.40482 (12)	1.32335 (11)	0.08442 (12)	0.0353 (3)	
C2	0.41364 (11)	1.20805 (11)	0.13210 (10)	0.0283 (3)	
C3	0.34903 (11)	1.12216 (11)	0.06975 (10)	0.0284 (3)	
H23	0.2999	1.1396	−0.0020	0.034*	
C4	0.35277 (10)	1.01170 (11)	0.10740 (10)	0.0267 (3)	
C5	0.42442 (11)	0.98674 (11)	0.21369 (10)	0.0280 (3)	
C6	0.48890 (11)	1.07419 (11)	0.27540 (10)	0.0310 (3)	
H4	0.5373	1.0571	0.3476	0.037*	
C7	0.48699 (11)	1.18407 (11)	0.23842 (10)	0.0297 (3)	
C8	0.56520 (13)	1.26927 (13)	0.31130 (12)	0.0412 (4)	
H1	0.6091	1.2331	0.3806	0.062*	
H2	0.5156	1.3295	0.3310	0.062*	
H3	0.6217	1.3005	0.2696	0.062*	
C9	0.43809 (13)	0.86813 (12)	0.26390 (11)	0.0358 (4)	
C10	0.33564 (19)	0.79143 (16)	0.19751 (16)	0.0364 (5)	0.744 (3)
H16	0.2599	0.8165	0.2176	0.044*	0.744 (3)
C11	0.32069 (18)	0.80706 (15)	0.07020 (16)	0.0352 (4)	0.744 (3)
H11	0.3979	0.7929	0.0493	0.042*	0.744 (3)
H12	0.2627	0.7515	0.0302	0.042*	0.744 (3)
C15	0.3518 (4)	0.6672 (3)	0.2281 (5)	0.0516 (9)	0.744 (3)
H13	0.2785	0.6265	0.1949	0.077*	0.744 (3)
H14	0.3692	0.6587	0.3106	0.077*	0.744 (3)
H15	0.4180	0.6368	0.1984	0.077*	0.744 (3)
C10B	0.3875 (6)	0.7792 (5)	0.1773 (5)	0.0364 (5)	0.256 (3)
H16B	0.4408	0.7776	0.1222	0.044*	0.256 (3)
C11B	0.2689 (5)	0.8159 (4)	0.1118 (5)	0.0352 (4)	0.256 (3)
H11B	0.2177	0.8350	0.1651	0.042*	0.256 (3)
H12B	0.2307	0.7532	0.0638	0.042*	0.256 (3)
C15B	0.3853 (14)	0.6606 (11)	0.2212 (16)	0.0516 (9)	0.256 (3)
H13B	0.4661	0.6383	0.2595	0.077*	0.256 (3)
H14B	0.3559	0.6098	0.1577	0.077*	0.256 (3)
H15B	0.3327	0.6569	0.2747	0.077*	0.256 (3)
C12	0.27785 (11)	0.92416 (11)	0.03173 (11)	0.0318 (3)	
C13	0.1469 (4)	0.9422 (4)	0.0305 (4)	0.0414 (9)	0.744 (3)
H5	0.0990	0.8866	−0.0190	0.062*	0.744 (3)
H6	0.1232	1.0176	0.0023	0.062*	0.744 (3)
H7	0.1339	0.9342	0.1075	0.062*	0.744 (3)
C14	0.2948 (2)	0.93749 (19)	−0.09371 (18)	0.0374 (5)	0.744 (3)
H8	0.3800	0.9388	−0.0936	0.056*	0.744 (3)
H9	0.2580	1.0076	−0.1258	0.056*	0.744 (3)
H10	0.2570	0.8744	−0.1395	0.056*	0.744 (3)
C16	0.4257 (4)	0.8693 (6)	0.3874 (3)	0.0576 (11)	0.744 (3)
H17	0.4953	0.9059	0.4345	0.086*	0.744 (3)
H18	0.4202	0.7922	0.4135	0.086*	0.744 (3)
H19	0.3535	0.9104	0.3930	0.086*	0.744 (3)
C17	0.5617 (2)	0.8238 (2)	0.2576 (2)	0.0464 (6)	0.744 (3)
H20	0.5676	0.8189	0.1782	0.070*	0.744 (3)
H21	0.5734	0.7494	0.2923	0.070*	0.744 (3)
H22	0.6230	0.8749	0.2982	0.070*	0.744 (3)
C13B	0.1450 (11)	0.9619 (14)	0.0068 (14)	0.0414 (9)	0.256 (3)
H5B	0.0939	0.8991	−0.0254	0.062*	0.256 (3)
H6B	0.1334	1.0242	−0.0473	0.062*	0.256 (3)
H7B	0.1241	0.9862	0.0773	0.062*	0.256 (3)
C14B	0.3189 (7)	0.8985 (6)	−0.0673 (6)	0.0374 (5)	0.256 (3)
H8B	0.3208	0.9670	−0.1115	0.056*	0.256 (3)
H9B	0.2652	0.8441	−0.1128	0.056*	0.256 (3)
H10B	0.3993	0.8668	−0.0462	0.056*	0.256 (3)
C16B	0.3894 (15)	0.876 (2)	0.3749 (13)	0.0576 (11)	0.256 (3)
H17B	0.4310	0.9355	0.4231	0.086*	0.256 (3)
H18B	0.4029	0.8042	0.4155	0.086*	0.256 (3)
H19B	0.3038	0.8918	0.3554	0.086*	0.256 (3)
C17B	0.5738 (8)	0.8426 (8)	0.3014 (7)	0.0464 (6)	0.256 (3)
H20B	0.6116	0.8534	0.2371	0.070*	0.256 (3)
H21B	0.5851	0.7651	0.3279	0.070*	0.256 (3)
H22B	0.6101	0.8934	0.3629	0.070*	0.256 (3)
C18	0.33250 (17)	1.43507 (14)	−0.07628 (16)	0.0561 (5)	
H24	0.2983	1.4873	−0.0297	0.084*	
H25	0.2796	1.4293	−0.1514	0.084*	
H26	0.4107	1.4624	−0.0838	0.084*	

### Atomic displacement parameters (Å^2^)

**Table:**

	*U*^11^	*U*^22^	*U*^33^	*U*^12^	*U*^13^	*U*^23^
O1	0.0551 (7)	0.0345 (6)	0.0423 (6)	−0.0050 (5)	0.0009 (5)	0.0096 (4)
O2	0.1106 (12)	0.0323 (6)	0.0585 (8)	−0.0166 (7)	−0.0076 (7)	0.0010 (5)
C1	0.0362 (7)	0.0302 (7)	0.0399 (7)	−0.0030 (5)	0.0091 (6)	0.0000 (5)
C2	0.0269 (6)	0.0288 (7)	0.0313 (6)	0.0003 (5)	0.0108 (5)	−0.0009 (5)
C3	0.0272 (6)	0.0312 (7)	0.0270 (6)	0.0007 (5)	0.0066 (5)	−0.0006 (5)
C4	0.0252 (6)	0.0293 (7)	0.0275 (6)	0.0000 (5)	0.0101 (5)	−0.0018 (5)
C5	0.0292 (6)	0.0312 (7)	0.0268 (6)	0.0023 (5)	0.0130 (5)	0.0012 (5)
C6	0.0320 (7)	0.0372 (7)	0.0243 (6)	0.0022 (5)	0.0072 (5)	−0.0009 (5)
C7	0.0277 (6)	0.0342 (7)	0.0289 (6)	−0.0004 (5)	0.0101 (5)	−0.0066 (5)
C8	0.0415 (8)	0.0406 (8)	0.0388 (7)	−0.0035 (6)	0.0031 (6)	−0.0098 (6)
C9	0.0452 (8)	0.0337 (7)	0.0306 (7)	0.0020 (6)	0.0132 (5)	0.0062 (5)
C10	0.0429 (12)	0.0309 (9)	0.0411 (10)	−0.0002 (9)	0.0216 (8)	0.0049 (7)
C11	0.0417 (10)	0.0276 (9)	0.0390 (10)	−0.0032 (7)	0.0149 (7)	−0.0046 (7)
C15	0.066 (3)	0.0331 (10)	0.0607 (13)	−0.0013 (14)	0.0255 (19)	0.0079 (8)
C10B	0.0429 (12)	0.0309 (9)	0.0411 (10)	−0.0002 (9)	0.0216 (8)	0.0049 (7)
C11B	0.0417 (10)	0.0276 (9)	0.0390 (10)	−0.0032 (7)	0.0149 (7)	−0.0046 (7)
C15B	0.066 (3)	0.0331 (10)	0.0607 (13)	−0.0013 (14)	0.0255 (19)	0.0079 (8)
C12	0.0322 (7)	0.0292 (7)	0.0340 (7)	−0.0025 (5)	0.0073 (5)	−0.0034 (5)
C13	0.0314 (8)	0.043 (2)	0.052 (2)	−0.0091 (10)	0.0133 (11)	−0.0071 (14)
C14	0.0466 (13)	0.0345 (13)	0.0325 (11)	−0.0042 (9)	0.0117 (9)	−0.0053 (8)
C16	0.091 (3)	0.0511 (14)	0.0374 (14)	0.006 (3)	0.029 (2)	0.0144 (12)
C17	0.0529 (12)	0.0383 (13)	0.0484 (16)	0.0109 (9)	0.0119 (13)	0.0057 (12)
C13B	0.0314 (8)	0.043 (2)	0.052 (2)	−0.0091 (10)	0.0133 (11)	−0.0071 (14)
C14B	0.0466 (13)	0.0345 (13)	0.0325 (11)	−0.0042 (9)	0.0117 (9)	−0.0053 (8)
C16B	0.091 (3)	0.0511 (14)	0.0374 (14)	0.006 (3)	0.029 (2)	0.0144 (12)
C17B	0.0529 (12)	0.0383 (13)	0.0484 (16)	0.0109 (9)	0.0119 (13)	0.0057 (12)
C18	0.0646 (11)	0.0412 (9)	0.0583 (10)	−0.0050 (8)	0.0038 (8)	0.0200 (7)

### Geometric parameters (Å, °)

**Table:**

O1—C1	1.3330 (17)	C11B—H11B	0.9900
O1—C18	1.4422 (18)	C11B—H12B	0.9900
O2—C1	1.1986 (18)	C15B—H13B	0.9800
C1—C2	1.4886 (18)	C15B—H14B	0.9800
C2—C3	1.3895 (18)	C15B—H15B	0.9800
C2—C7	1.4060 (18)	C12—C14B	1.415 (7)
C3—C4	1.3939 (18)	C12—C13	1.519 (4)
C3—H23	0.9500	C12—C13B	1.559 (12)
C4—C5	1.4005 (18)	C12—C14	1.581 (2)
C4—C12	1.5270 (17)	C13—H5	0.9800
C5—C6	1.3994 (18)	C13—H6	0.9800
C5—C9	1.5365 (18)	C13—H7	0.9800
C6—C7	1.3858 (19)	C14—H8	0.9800
C6—H4	0.9500	C14—H9	0.9800
C7—C8	1.5092 (18)	C14—H10	0.9800
C8—H1	0.9800	C16—H17	0.9800
C8—H2	0.9800	C16—H18	0.9800
C8—H3	0.9800	C16—H19	0.9800
C9—C10B	1.517 (6)	C17—H20	0.9800
C9—C16	1.532 (4)	C17—H21	0.9800
C9—C17	1.535 (3)	C17—H22	0.9800
C9—C17B	1.558 (9)	C13B—H5B	0.9800
C9—C16B	1.567 (12)	C13B—H6B	0.9800
C9—C10	1.569 (2)	C13B—H7B	0.9800
C10—C11	1.525 (3)	C14B—H8B	0.9800
C10—C15	1.531 (4)	C14B—H9B	0.9800
C10—H16	1.0000	C14B—H10B	0.9800
C11—C12	1.522 (2)	C16B—H17B	0.9800
C11—H11	0.9900	C16B—H18B	0.9800
C11—H12	0.9900	C16B—H19B	0.9800
C15—H13	0.9800	C17B—H20B	0.9800
C15—H14	0.9800	C17B—H21B	0.9800
C15—H15	0.9800	C17B—H22B	0.9800
C10B—C11B	1.487 (8)	C18—H24	0.9800
C10B—C15B	1.515 (13)	C18—H25	0.9800
C10B—H16B	1.0000	C18—H26	0.9800
C11B—C12	1.633 (5)		
			
C1—O1—C18	116.18 (12)	C12—C11B—H12B	109.1
O2—C1—O1	121.91 (13)	H11B—C11B—H12B	107.9
O2—C1—C2	125.66 (13)	C10B—C15B—H13B	109.5
O1—C1—C2	112.42 (11)	C10B—C15B—H14B	109.5
C3—C2—C7	119.33 (12)	H13B—C15B—H14B	109.5
C3—C2—C1	119.32 (11)	C10B—C15B—H15B	109.5
C7—C2—C1	121.35 (12)	H13B—C15B—H15B	109.5
C2—C3—C4	123.16 (11)	H14B—C15B—H15B	109.5
C2—C3—H23	118.4	C14B—C12—C13	122.5 (4)
C4—C3—H23	118.4	C14B—C12—C11	85.1 (3)
C3—C4—C5	118.17 (11)	C13—C12—C11	112.82 (19)
C3—C4—C12	118.64 (11)	C14B—C12—C4	114.0 (3)
C5—C4—C12	123.19 (12)	C13—C12—C4	109.7 (2)
C6—C5—C4	117.95 (12)	C11—C12—C4	110.08 (12)
C6—C5—C9	118.74 (11)	C14B—C12—C13B	113.2 (7)
C4—C5—C9	123.29 (12)	C4—C12—C13B	108.6 (7)
C7—C6—C5	124.45 (11)	C13—C12—C14	107.86 (18)
C7—C6—H4	117.8	C11—C12—C14	106.67 (13)
C5—C6—H4	117.8	C4—C12—C14	109.60 (12)
C6—C7—C2	116.93 (11)	C13B—C12—C14	96.1 (6)
C6—C7—C8	119.00 (12)	C14B—C12—C11B	114.1 (4)
C2—C7—C8	124.04 (12)	C13—C12—C11B	85.8 (2)
C7—C8—H1	109.5	C4—C12—C11B	106.7 (2)
C7—C8—H2	109.5	C13B—C12—C11B	99.1 (6)
H1—C8—H2	109.5	C14—C12—C11B	133.4 (2)
C7—C8—H3	109.5	C12—C13—H5	109.5
H1—C8—H3	109.5	C12—C13—H6	109.5
H2—C8—H3	109.5	C12—C13—H7	109.5
C10B—C9—C16	125.2 (4)	C12—C14—H8	109.5
C10B—C9—C17	86.8 (3)	C12—C14—H9	109.5
C16—C9—C17	109.67 (19)	C12—C14—H10	109.5
C10B—C9—C5	112.5 (2)	C9—C16—H17	109.5
C16—C9—C5	110.8 (3)	C9—C16—H18	109.5
C17—C9—C5	108.32 (14)	C9—C16—H19	109.5
C10B—C9—C17B	105.9 (4)	C9—C17—H20	109.5
C16—C9—C17B	91.1 (4)	C9—C17—H21	109.5
C5—C9—C17B	107.8 (4)	C9—C17—H22	109.5
C10B—C9—C16B	118.5 (10)	C12—C13B—H5B	109.5
C17—C9—C16B	124.8 (6)	C12—C13B—H6B	109.5
C5—C9—C16B	105.2 (9)	H5B—C13B—H6B	109.5
C17B—C9—C16B	106.5 (7)	C12—C13B—H7B	109.5
C17—C9—C10	111.94 (15)	H5B—C13B—H7B	109.5
C5—C9—C10	109.60 (12)	H6B—C13B—H7B	109.5
C17B—C9—C10	128.9 (4)	C12—C14B—H8B	109.5
C16B—C9—C10	95.9 (9)	C12—C14B—H9B	109.5
C11—C10—C15	110.1 (2)	H8B—C14B—H9B	109.5
C11—C10—C9	110.65 (14)	C12—C14B—H10B	109.5
C15—C10—C9	113.8 (2)	H8B—C14B—H10B	109.5
C11—C10—H16	107.3	H9B—C14B—H10B	109.5
C15—C10—H16	107.3	C9—C16B—H17B	109.5
C9—C10—H16	107.3	C9—C16B—H18B	109.5
C12—C11—C10	112.30 (14)	H17B—C16B—H18B	109.5
C12—C11—H11	109.1	C9—C16B—H19B	109.5
C10—C11—H11	109.1	H17B—C16B—H19B	109.5
C12—C11—H12	109.1	H18B—C16B—H19B	109.5
C10—C11—H12	109.1	C9—C17B—H20B	109.5
H11—C11—H12	107.9	C9—C17B—H21B	109.5
C11B—C10B—C15B	112.3 (8)	H20B—C17B—H21B	109.5
C11B—C10B—C9	109.3 (4)	C9—C17B—H22B	109.5
C15B—C10B—C9	116.5 (9)	H20B—C17B—H22B	109.5
C11B—C10B—H16B	106.0	H21B—C17B—H22B	109.5
C15B—C10B—H16B	106.0	O1—C18—H24	109.5
C9—C10B—H16B	106.0	O1—C18—H25	109.5
C10B—C11B—C12	112.4 (4)	H24—C18—H25	109.5
C10B—C11B—H11B	109.1	O1—C18—H26	109.5
C12—C11B—H11B	109.1	H24—C18—H26	109.5
C10B—C11B—H12B	109.1	H25—C18—H26	109.5
			
C18—O1—C1—O2	1.2 (2)	C17B—C9—C10—C15	36.1 (5)
C18—O1—C1—C2	179.86 (13)	C16B—C9—C10—C15	−80.7 (8)
O2—C1—C2—C3	169.86 (15)	C15—C10—C11—C12	167.6 (2)
O1—C1—C2—C3	−8.72 (18)	C9—C10—C11—C12	−65.6 (2)
O2—C1—C2—C7	−10.8 (2)	C16—C9—C10B—C11B	94.2 (5)
O1—C1—C2—C7	170.60 (11)	C17—C9—C10B—C11B	−153.9 (4)
C7—C2—C3—C4	0.10 (18)	C5—C9—C10B—C11B	−45.4 (5)
C1—C2—C3—C4	179.43 (11)	C17B—C9—C10B—C11B	−162.8 (5)
C2—C3—C4—C5	0.59 (18)	C16B—C9—C10B—C11B	77.8 (9)
C2—C3—C4—C12	179.94 (11)	C10—C9—C10B—C11B	43.7 (4)
C3—C4—C5—C6	−0.56 (17)	C16—C9—C10B—C15B	−34.4 (8)
C12—C4—C5—C6	−179.88 (11)	C17—C9—C10B—C15B	77.5 (7)
C3—C4—C5—C9	−178.83 (11)	C5—C9—C10B—C15B	−174.0 (6)
C12—C4—C5—C9	1.85 (18)	C17B—C9—C10B—C15B	68.5 (8)
C4—C5—C6—C7	−0.15 (19)	C16B—C9—C10B—C15B	−50.9 (10)
C9—C5—C6—C7	178.20 (11)	C10—C9—C10B—C15B	−85.0 (8)
C5—C6—C7—C2	0.83 (19)	C15B—C10B—C11B—C12	−161.7 (8)
C5—C6—C7—C8	−177.28 (12)	C9—C10B—C11B—C12	67.4 (5)
C3—C2—C7—C6	−0.78 (17)	C10—C11—C12—C14B	162.2 (4)
C1—C2—C7—C6	179.90 (11)	C10—C11—C12—C13	−74.5 (3)
C3—C2—C7—C8	177.23 (12)	C10—C11—C12—C4	48.37 (18)
C1—C2—C7—C8	−2.10 (19)	C10—C11—C12—C13B	−83.0 (8)
C6—C5—C9—C10B	−166.3 (3)	C10—C11—C12—C14	167.20 (16)
C4—C5—C9—C10B	12.0 (3)	C10—C11—C12—C11B	−41.0 (4)
C6—C5—C9—C16	48.2 (2)	C3—C4—C12—C14B	69.9 (3)
C4—C5—C9—C16	−133.5 (2)	C5—C4—C12—C14B	−110.8 (3)
C6—C5—C9—C17	−72.09 (16)	C3—C4—C12—C13	−71.72 (18)
C4—C5—C9—C17	106.17 (16)	C5—C4—C12—C13	107.60 (17)
C6—C5—C9—C17B	−50.0 (3)	C3—C4—C12—C11	163.56 (12)
C4—C5—C9—C17B	128.3 (3)	C5—C4—C12—C11	−17.12 (17)
C6—C5—C9—C16B	63.4 (8)	C3—C4—C12—C13B	−57.3 (6)
C4—C5—C9—C16B	−118.4 (8)	C5—C4—C12—C13B	122.0 (6)
C6—C5—C9—C10	165.52 (12)	C3—C4—C12—C14	46.53 (17)
C4—C5—C9—C10	−16.23 (17)	C5—C4—C12—C14	−134.15 (14)
C16—C9—C10—C11	166.1 (2)	C3—C4—C12—C11B	−163.2 (2)
C17—C9—C10—C11	−74.04 (19)	C5—C4—C12—C11B	16.1 (3)
C5—C9—C10—C11	46.17 (18)	C10B—C11B—C12—C14B	75.9 (6)
C17B—C9—C10—C11	−88.6 (5)	C10B—C11B—C12—C13	−160.2 (5)
C16B—C9—C10—C11	154.6 (7)	C10B—C11B—C12—C11	50.5 (4)
C16—C9—C10—C15	−69.3 (3)	C10B—C11B—C12—C4	−50.9 (5)
C17—C9—C10—C15	50.6 (2)	C10B—C11B—C12—C13B	−163.5 (8)
C5—C9—C10—C15	170.84 (18)	C10B—C11B—C12—C14	89.0 (5)

### Hydrogen-bond geometry (Å, °)

**Table:**

*D*—H···*A*	*D*—H	H···*A*	*D*···*A*	*D*—H···*A*
C3—H23···O1	0.95	2.32	2.6839 (17)	103
C18—H26···O2^i^	0.98	2.47	3.397 (2)	157

Symmetry codes: (i) −*x*+1, −*y*+3, −*z*.
